# Fabry disease presenting as bilateral medial medullary infarction with a “heart appearance” sign: a case report

**DOI:** 10.1186/s12883-020-01766-5

**Published:** 2020-05-12

**Authors:** Shuai Jiang, Lei Wang, Yuying Yan, Qiange Zhu, Jincheng Wan, Jiayu Sun, Bo Wu

**Affiliations:** 1 Department of Neurology, West China Hospital, Sichuan University, 37 Guo Xue Xiang, Chengdu, 610041 China; 2 Department of Neurology, The Third People’s Hospital of Chengdu, Chengdu, China; 3 Department of Radiology, West China Hospital, Sichuan University, Chengdu, China

**Keywords:** Fabry disease, Bilateral medial medullary infarction, Cryptogenic stroke, Case report

## Abstract

**Background:**

The etiologic determinants of cryptogenic stroke remain a diagnostic challenge in clinical practice. Fabry disease (FD) is one of the monogenic causes of stroke that may remain unrecognized as a potential contributing causative factor, because of its rarity and difficulty in diagnosis. We report a case with rare bilateral medial medullary infarction manifesting as “heart appearance” who was diagnosed with FD.

**Case presentation:**

A 51-year-old Chinese man presented with acute dysarthria and mild tetraparesis. In the 24 h following admission, the patient rapidly developed progressive flaccid quadriplegia and tongue weakness, necessitating ventilator support. Immediate magnetic resonance imaging of the brain showed heart-shaped appearance of bilateral medial medullary infarction. The patient suffered two new subcortical infarcts 40 days after the first. Detailed Family history and physical examination indicated symptoms consistent with FD, which was confirmed by very low alpha galactosidase A levels and a missense mutation of the alpha-galactosidase A gene**.**

**Conclusions:**

We report what appears to be the first case of FD manifesting as bilateral medial medullary infarction. Our case suggests that clinicians should consider the possibility of FD in patients with cryptogenic stroke, especially when combined with infarction in the vertebrobasilar artery system, renal insufficiency, or cardiomyopathy. A detailed analysis of subtle historical clues and performing a complete physical examination on stroke patients would help promote earlier diagnosis of FD.

## Background

Fabry disease (FD) is a rare multisystemic X-linked lysosomal storage disorder associated with renal, cardiac, and cerebrovascular complications [1]. The incidence of Fabry’s disease has been estimated to be approximately 1 in 40,000 to 1 in 117,000 in the general population [2]. Due to the non-specificity of initial symptoms and rarity of the disease, many patients are misdiagnosed or diagnosed later in life.

Early-onset ischemic stroke, predominantly in the posterior circulation, is an important feature of FD [3]. Medial medullary infarction (MMI) accounts for 0.5–1.5% of all brain infarctions [4, 5]. Very rarely, MMI can occur bilaterally, resulting in a unique “heart appearance” on magnetic resonance imaging (MRI). However, we are unaware of FD cases involving bilateral MMI. Here we report a case of a man with bilateral MMI manifesting as “heart appearance” who was diagnosed with FD.

## Case presentation

A 51-year-old man presented at our hospital with acute dysarthria and mild tetraparesis since 3 days in May 2017. He had bilateral hearing loss, for which he had been using hearing aids from the age of 40. His sister had died suddenly of unknown causes at the age of 36. No other cardiovascular risk factors were found. On admission, physical examination revealed bilateral horizontal gaze-evoked nystagmus, mild dysarthria, and decreased pharyngeal reflex. The test for bilateral Babinski’s reflex was positive. An urgent computed tomography (CT) scan showed chronic lacunar infarction in bilateral centrum semiovale. In the 24 h following admission, the patient rapidly developed progressive flaccid quadriplegia and tongue weakness, necessitating ventilator support. The plantar response became absent bilaterally.

**Fig.1:**
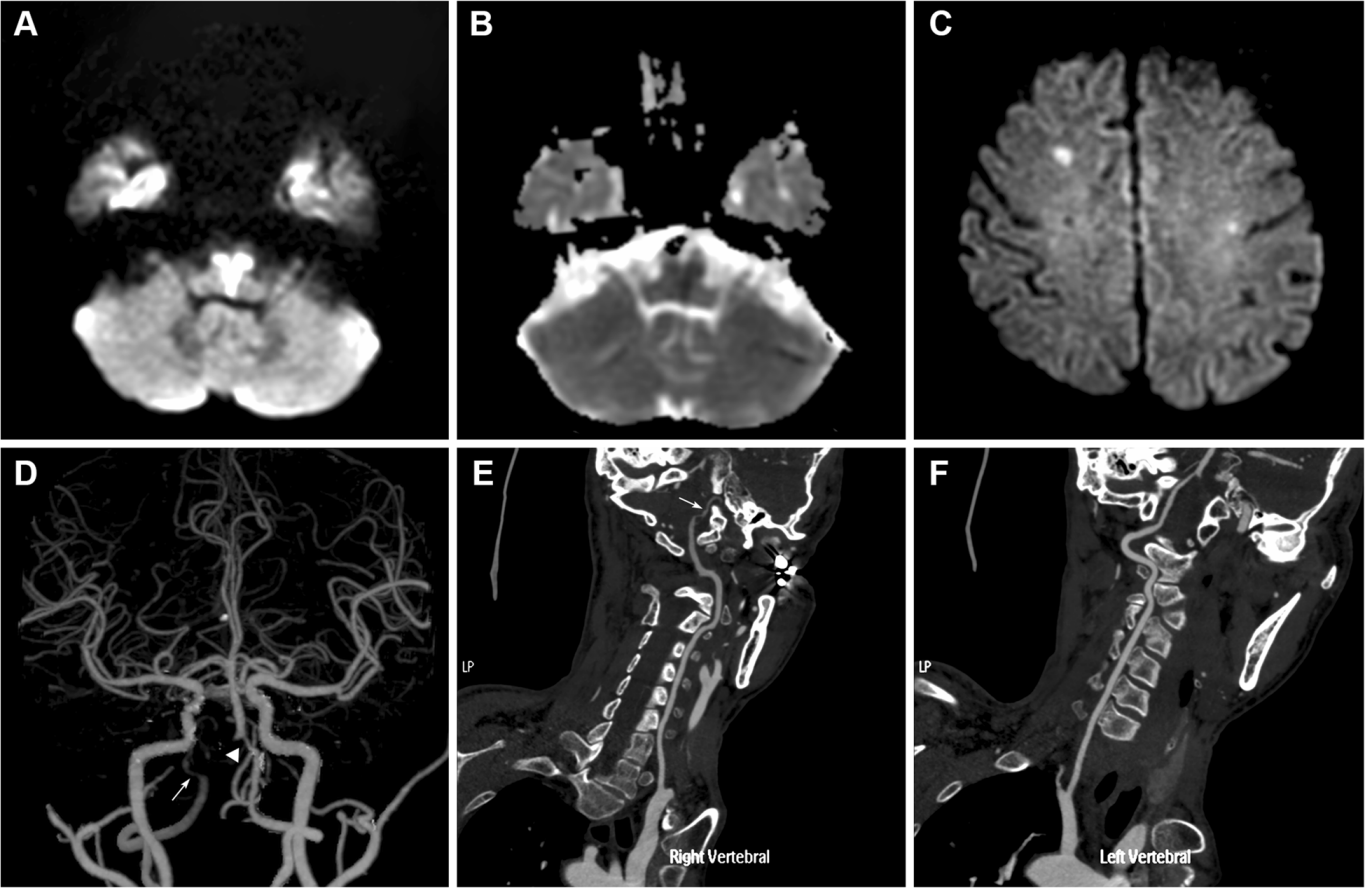
**a** Brain diffusion-weighted image on admission showing a “heart-shaped” hyperintense area in the bilateral ventral medulla, characteristic of bilateral MMI. **b** Corresponding apparent diffusion coefficient map, showing restricted diffusion within the heart-shaped lesion. **c** Diffusion-weighted image at 40 days after admission showing two small subcortical infarcts in bilateral cerebral hemisphere. **d-f** Computed tomography angiogram showing mild stenosis in the left vertebrobasilar junction (arrowhead) and anatomic variability of the right vertebral artery, which extended directly into the posterior inferior cerebellar artery (arrows)

**Fig.2:**
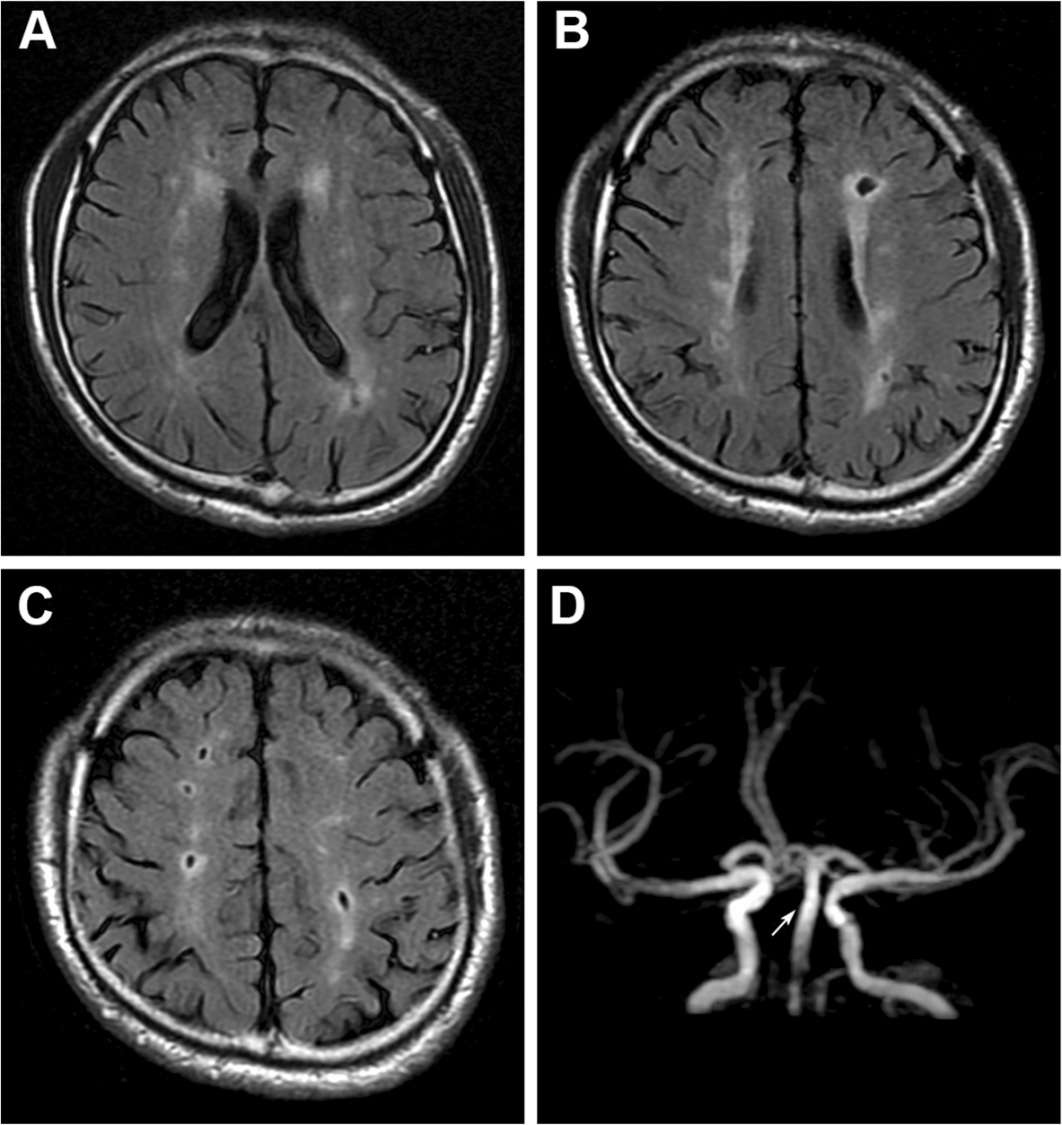
**a-c** Axial fluid attenuated inversion recovery images showing multiple white matter hyperintensities and old lacunar strokes in bilateral cerebral hemispheres. **d** Magnetic resonance angiography showing mild to moderate ectasia of the basilar artery (arrows)

For the possibility of Guillain-Barre syndrome and brainstem encephalitis, lumbar puncture was performed which showed normal. Subsequently, an emergent brain diffusion-weighted imaging revealed a heart-shaped area of hyperintensity in the bilateral ventral medulla (Fig. 1a) with apparent diffusion coefficient reversal (Fig. 1b). Fluid-attenuated inversion recovery (FLAIR) detected multiple deep white matter hyperintensities and subcortical infarcts (Fig. 2a-c). Magnetic resonance angiography showed mild to moderate ectasia of the basilar artery (Fig. 2d). Computed tomographic angiography (CTA) revealed mild stenosis in the left vertebrobasilar junction and the right vertebral artery, which did not converge with the proximal basilar artery (Fig. 1d-e). Electrocardiography revealed sinus bradyarrhythmia, and an echocardiogram showed concentric left ventricular hypertrophy with left ventricle outflow tract obstruction. Serum creatinine levels were within the normal range, and urine protein levels were mild increase (781.8 mg/24 h).

**Fig.3:**
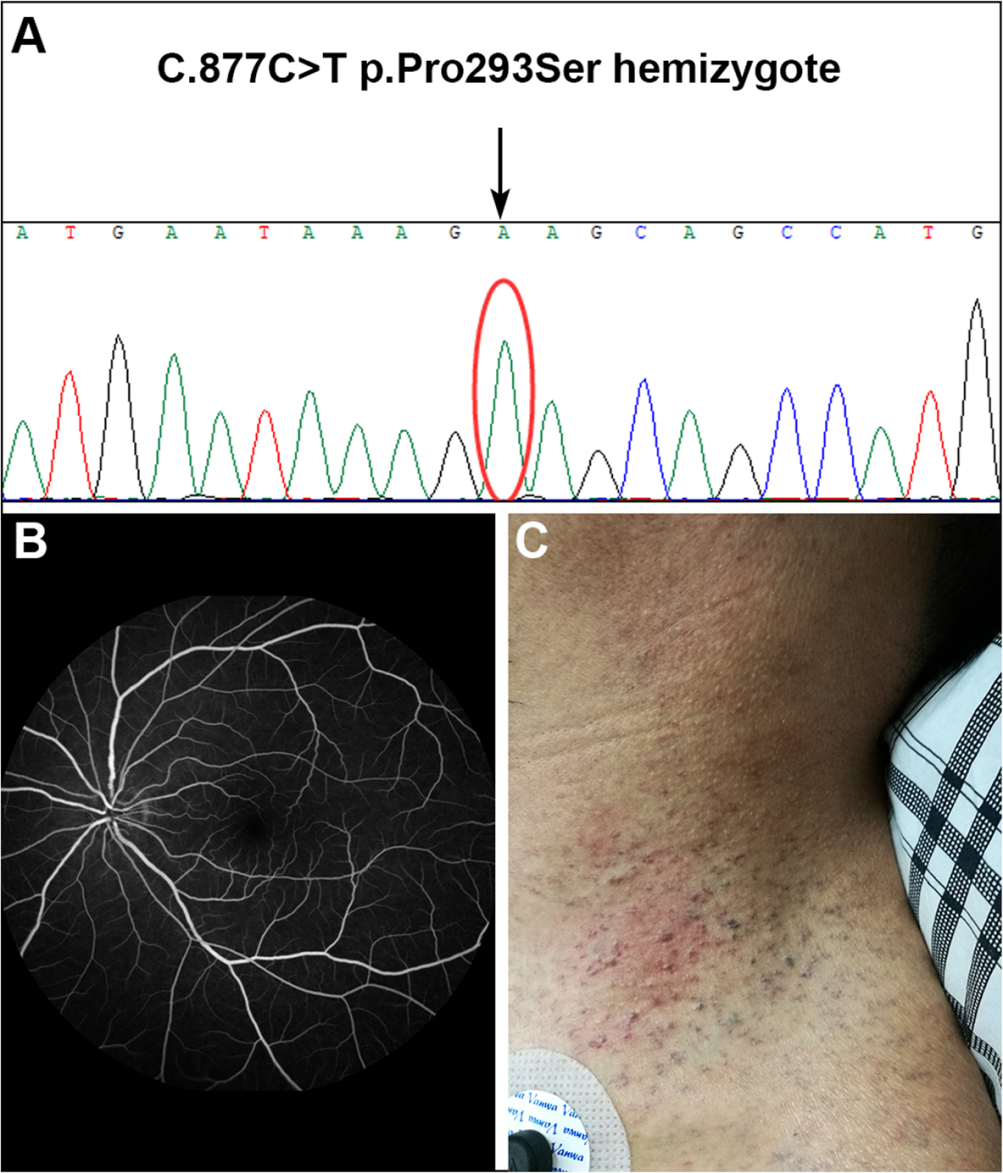
**a** Alignment of gene sequences. Portion of the sequence of exon 6 of the *GLA* gene in the patient. The red circle indicates the position of the mutation c.877C > T [p.P293S]. **b** Fluorescein angiography of the fundus, showing increased tortuosity of retinal arteries and veins. **c** Reddish-purple skin lesions (angiokeratomas) on the neck, characteristic of Fabry disease

At 40 days after admission, unexpectedly, a scheduled repeat diffusion-weighted scan revealed two new subcortical infarcts in the bilateral cerebral hemispheres (Fig. 1c). We first considered the possibility of a cardioembolic cause due to the hypertrophic cardiomyopathy. However, detailed analysis of the patient’s history revealed typical clinical manifestations of FD: painful acroparesthesias, hypohidrosis, and postprandial diarrhea from the age of 6. Angiokeratomas were also found in the lower abdomen, groin, and neck (Fig. 3c). Ophthalmological evaluation showed increased tortuosity of the retinal vessels (Fig. 3b). His mother had hypertrophic cardiomyopathy. His eldest daughter and second daughter had mild episodic burning pain in the limbs since age 6 and age 9, respectively, and the second daughter had shown skin lesions similar to the patient on her back starting from age 12. The remaining third daughter and youngest son reported no clinical symptoms and signs**.** The leukocyte α-galactosidase A activity was found to be dramatically reduced (0.1 nmol/day/spot; normal: 100–500). FD diagnosis was confirmed by sequencing the α-galactosidase A gene (Fig. 3a), which revealed the previously described c.877C > T [p.P293S] missense mutation [6]. Genetic analysis in families revealed his mother and three daughters had the same GLA gene mutation in heterozygosis**.** The patient declined enzyme replacement therapy because of the high cost. He was treated with aspirin and atorvastatin and was extubated after 3 weeks. After 2 months of treatment and rehabilitation, the patient could sit in a wheel chair but his motor function did not recover to a usable level**.** Despite the standard ischemic stroke prevention measures, follow-up showed the patient died of heart failure 2 years later.

## Discussion and conclusions

Here we report the first known case of FD manifesting as bilateral MMI. Patients with FD are known to be at increased risk of cerebrovascular complications, which predominantly affects circulation in the posterior circulatory system [3]. In the large cohort of the Fabry Registry (*n* = 2446), the prevalence of strokes in FD was estimated to be 6.9% in males and 4.3% in females, much higher than in the general population [7]. However, to our knowledge, the extremely rare stroke subtype of bilateral MMI has never been reported in FD.

Bilateral MMI is a rare stroke subtype and manifests as a characteristic “heart appearance” or “V-shaped” sign on MRI [4, 8]. According to vascular supply, the medulla oblongata is divided into anteromedial, anterolateral, lateral and posterior territory [9]. The medial medullary territory (anterior-medial territory and anterior-lateral) is supplied by penetrating arterioles from the anterior spinal artery and vertebral artery [5]. Infarction involving bilateral anterior-medial territory and the anterior-lateral territory can result in a “heart appearance” sign. Misdiagnosis or delay in diagnosis in this syndrome is common as patient presenting with progressive weakness with diffuse areflexia may be misdiagnosed with Guillain-Barre syndrome or its variants [10]. Therefore, immediate lumbar puncture and MRI scan are essential to make a definite diagnosis in the early stages of bilateral MMI. The etiology mechanism of bilateral MMI involves vertebral artery atherosclerosis, atheromatous branch occlusion, cardioembolism, and dissection of vertebral artery [4]. The occurrence of a simultaneous bilateral MMI is thought to be related to the occlusion of anatomic variability branches originating from a vertebral artery that supply both sides of the medial medullary [4], which is consistent with the finding of our patient (Fig. 1d-e). Since no other atherosclerotic risk factors or arterial dissection were found in this patient, the present case raises the possibility that the aberrant vascular morphological changes in the vertebrobasilar system are associated with FD.

FD is a rare lysosomal storage disorder caused by the mutations of α-galactosidase A gene, which leads to a deficiency of α-galactosidase A. The resultant accumulation of globotriaosylceramide (Gb3) in multiple cell types and tissues promotes development of disease-related complications associated with renal, cardiovascular, and cerebrovascular involvement [2]. Progressive accumulations of Gb3 occur in endothelial cells and smooth muscle cells of cerebral vessels, leading to progressive stenosis and occlusion as well as dilatation of blood vessels, which involve both large and small arteries of the brain [11]. Vascular ectasia occurs more frequently in the posterior circulation, and vertebrobasilar dolichoectasia could serve as an early marker of FD [12]. Small-vessel disease is more commonly seen in patients with FD [3].White matter hyperintensities are thought to represent small vessel disease in FD cerebral vasculopathy [13], as we observed in our patient (Fig. 2a-c). The etiologies of ischemic stroke in FD can be attributed to the direct consequence of cardiogenic embolism or a combination of changes in both large and small vessel walls secondary to glycolipid accumulation and abnormal cerebrovascular autoregulation and vasoreactivity, and a prothrombotic state with likely activation of reactive oxygen species [14, 15].

Typical manifestations of FD include neuropathic pain, cutaneous angiokeratomas, anhidrosis, gastrointestinal symptoms, cornea verticillate, proteinuria, and progressive impairment of renal and cardiac functions [16]. Since FD affects multiple organ systems and its symptoms are non-specific, it is common to have long delays between symptom onset and diagnosis. This was the case for our patient: despite exhibiting multiple features typical of the disease for at least 45 years, he remained undiagnosed until he developed severe bilateral MMI. Previous studies have been found FD in 0.9 to 4% of young patients with cryptogenic stroke [17–20]. Thus, we propose that clinicians consider the possibility of FD in cases of cryptogenic stroke, especially when combined with infarction in the vertebrobasilar artery system, renal insufficiency, or cardiomyopathy.

As hypertrophic cardiomyopathy is a cardiovascular risk factor for stroke, cardiogenic embolism was vulnerable to be considered as the final diagnosis based on his stroke topography, thus the potential intrinsic cause would be neglected. However, after careful evaluation of the patient’s typical clinical manifestations, his prominent concentric hypertrophic cardiomyopathy, early-onset hearing loss, and family history were all suggestive of FD. Thus, we also propose that including a detailed analysis of clinical history and performing a complete physical examination on stroke patients would help promote earlier diagnosis of this disorder. Earlier diagnosis of FD may provide patients with earlier access to effective therapies before they develop severe cerebrovascular damage.

## Data Availability

All data related to this case report are documented within this manuscript.

## Abbreviations

CT: Computed tomography
CTA: Computed tomographic angiography
FLAIR: Fluid-attenuated inversion recovery
MMI: Medial medullary infarction
MRI: magnetic resonance imaging

## References

[1] Viana-Baptista M (2012). Stroke and Fabry disease. J Neurol.

[2] Zarate YA, Hopkin RJ (2008). Fabry’s disease. Lancet (London, England).

[3] Kolodny E, Fellgiebel A, Hilz MJ, Sims K, Caruso P, Phan TG (2015). Cerebrovascular involvement in Fabry disease: current status of knowledge. Stroke..

[4] Pongmoragot J, Parthasarathy S, Selchen D, Saposnik G (2013). Bilateral medial medullary infarction: a systematic review. J Stroke Cerebrovasc Dis.

[5] Toyoda K, Imamura T, Saku Y, Oita J, Ibayashi S, Minematsu K (1996). Medial medullary infarction: analyses of eleven patients. Neurology..

[6] Riera C, Lois S, Dominguez C, Fernandez-Cadenas I, Montaner J, Rodriguez-Sureda V (2015). Molecular damage in Fabry disease: characterization and prediction of alpha-galactosidase a pathological mutations. Proteins..

[7] Sims K, Politei J, Banikazemi M, Lee P (2009). Stroke in Fabry disease frequently occurs before diagnosis and in the absence of other clinical events: natural history data from the Fabry registry. Stroke..

[8] Maeda M, Shimono T, Tsukahara H, Maier SE, Takeda K (2004). Acute bilateral medial medullary infarction: a unique ‘heart appearance’ sign by diffusion-weighted imaging. Eur Neurol.

[9] Bassetti C, Bogousslavsky J, Mattle H, Bernasconi A (1997). Medial medullary stroke: report of seven patients and review of the literature. Neurology..

[10] Lee E-S, Sung K-B, Lee T-K (2017). Teaching video NeuroImages: upbeat and horizontal gaze-evoked nystagmus in bilateral medial medullary infarction. Neurology..

[11] Crutchfield KE, Patronas NJ, Dambrosia JM, Frei KP, Banerjee TK, Barton NW (1998). Quantitative analysis of cerebral vasculopathy in patients with Fabry disease. Neurology..

[12] Fellgiebel A, Keller I, Marin D, Müller MJ, Schermuly I, Yakushev I (2009). Diagnostic utility of different MRI and MR angiography measures in Fabry disease. Neurology..

[13] Fellgiebel A, Müller MJ, Ginsberg L (2006). CNS manifestations of Fabry's disease. Lancet Neurol.

[14] Moore DF, Kaneski CR, Askari H, Schiffmann R (2007). The cerebral vasculopathy of Fabry disease. J Neurol Sci.

[15] Moore DF, Scott LT, Gladwin MT, Altarescu G, Kaneski C, Suzuki K (2001). Regional cerebral hyperperfusion and nitric oxide pathway dysregulation in Fabry disease: reversal by enzyme replacement therapy. Circulation..

[16] El-Abassi R, Singhal D, England JD (2014). Fabry's disease. J Neurol Sci.

[17] Fancellu L, Borsini W, Romani I, Pirisi A, Deiana GA, Sechi E (2015). Exploratory screening for Fabry’s disease in young adults with cerebrovascular disorders in northern Sardinia. BMC Neurol.

[18] Dubuc V, Moore DF, Gioia LC, Saposnik G, Selchen D, Lanthier S (2013). Prevalence of Fabry disease in young patients with cryptogenic ischemic stroke. J Stroke Cerebrovasc Dis.

[19] Rolfs A, Bottcher T, Zschiesche M, Morris P, Winchester B, Bauer P (2005). Prevalence of Fabry disease in patients with cryptogenic stroke: a prospective study. Lancet (London, England).

[20] Baptista MV, Ferreira S, Pinho-E-Melo T, Carvalho M, Cruz VT, Carmona C (2010). Mutations of the GLA gene in young patients with stroke: the PORTYSTROKE study--screening genetic conditions in Portuguese young stroke patients. Stroke..

